# Antioxidant Action and Therapeutic Efficacy of *Allium sativum* L.

**DOI:** 10.3390/molecules18010690

**Published:** 2013-01-04

**Authors:** Anna Capasso

**Affiliations:** Department of Pharmacy, University of Salerno, Via Ponte don Melillo, Fisciano (Salerno) 84084, Italy; E-Mail: annacap@unisa.it; Tel.: +39-089-969-744; Fax: +39-089-969-602

**Keywords:** *Allium*, garlic, antioxidant activity

## Abstract

*Allium sativum* (L.) is rich in antioxidants which help destroy free radicals particles that can damage cell membranes and DNA, and may contribute to the aging process as well as the development of a number of conditions, including heart disease and cancer. Antioxidants neutralize free radicals and may reduce or even help prevent some of the damage they cause over time. The antioxidant activity of fresh *Allium sativum* L. (garlic) is well known and is mainly due to unstable and irritating organosulphur compounds. Fresh garlic extracted over a prolonged period (up to 20 months) produces odourless aged garlic extract (AGE) containing stable and water soluble organosulphur compounds that prevent oxidative damage by scavenging free radicals. The aim of this review was to understand the mechanism of antioxidant action and therapeutic efficacy of garlic.

## 1. Introduction

**Figure 1:**
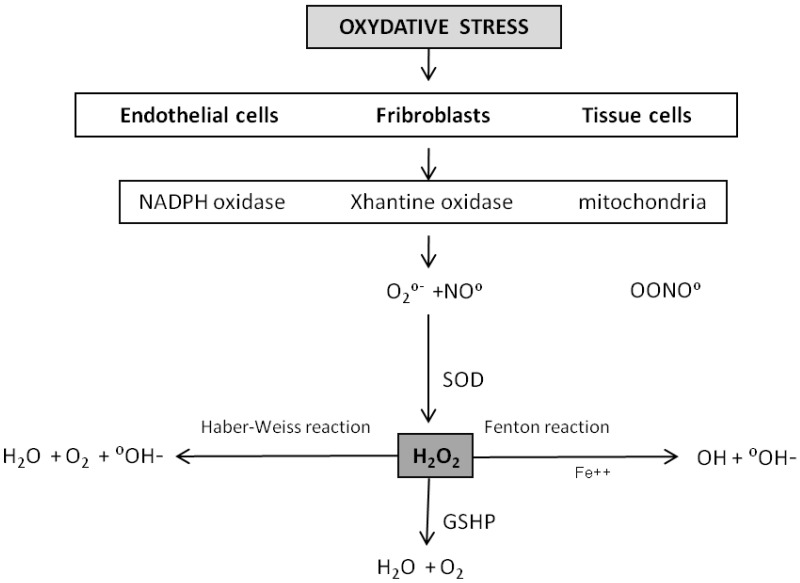
ROS origin and pathway.

The free radical theory is based on the evidence that living organisms (aerobes) produce oxygen-centered free radicals, capable of inducing irreversible damage to biological structures. These are formed inside body cells when oxygen is used in metabolic processes in order to produce energy. Mitochondrial respiration produces Reactive Oxygen Species (ROS) by leakage of intermediates from the electron transport chain [1]. These molecules are highly unstable because they have an unpaired electron; they therefore seek to achieve a stable state by appropriating electrons from nearby molecules, these in turn become unstable and so on, thus creating an instability chain reaction. Usually, the harmful activity of a small percentage of these free radicals is inhibited by the natural antioxidants occurring in the cell. Antioxidants may be divided into enzymatic (superoxide dismutase [SOD], catalase, glutathione peroxidase [GSHP]) and non-enzymatic (vitamins E, C, A) groups. When, however, the amount of free radical increases (due to overeating, smoking, drug abuse, ultraviolet radiation exposure, persistent chronic inflammation, *etc.*) the pool of antioxidants is saturated and the excess of free radicals damages biological structures. At first the damage is evident in mitochondria, which have the potential to affect the DNA and the RNA causing mutations, but also on proteins and lipids. Endothelial cells, fibroblasts and tissue cells are the most affected by oxidative stress. In order to understand how free radicals may be involved in ageing organs it is worth reviewing the production and fate of the ROS at the tissue level. Oxidases and mitochondria are the main ROS producers, which include highly reactive molecules such as superoxide anion (O^2−^). The fate of this molecule depends on the total oxidation-reduction equilibrium at the cellular level, which is supervised by the SODs which produce H_2_O_2_ (hydrogen peroxide) (2) which is typically converted into H_2_O and O_2_ through an enzymatic reaction catalysed by a catalase or the GSHP. When the production of superoxide anion or of H_2_O_2_ is such as to saturate the reductive capacity of the SODs or of the GSHP, these molecules become the substrate for the creation of highly reactive molecules such as hydroxyl (∞OH) (by means of a Fenton and/or Haber-Weiss reaction) which are responsible for cell and tissue damage [2]. The superoxide anion can react with nitric oxide in a diffusion limited reaction generating peroxynitrite, itself a powerful ROS. Most ROS are limited in their diffusion potential by their insolubility in lipids, and thus their effect is generally limited to the intracellular compartment of production. Conversely, H_2_O_2_ can get through cell membranes and thus react farther away from the production site. This theory submits that the mitochondrial production of ROS regulate the ageing rate since oxidative damage builds up over time. According to this hypothesis long-lived species produce fewer ROS by comparison to shorter-lived species, and mice subjected to calorie restriction live longer and produce fewer ROS than controls (Figure 1) [2].

Free radicals play an important role, in both health and disease, and have been implicated in manifold human disease processes. They are extremely reactive and unstable molecules that can damage cells and cell DNA leading to cell mutation and destruction [3]. Fortunately, organisms utilize both enzymatic and nonenzymatic endogenous antioxidant defenses to minimize cell injury. Nevertheless the reinforcement of endogenous antioxidant may be particularly important when free radical generation is enhanced. Antioxidants are naturally abundant in fruits and vegetables and are able to neutralize free radicals donating an electron and converting them to harmless molecules [4]. 

Garlic (*Allium sativum* L.) is one of the World’s oldest medicines and has been employed not only for flavouring, but also as a medical herb for its diverse biological activities, including anti-carcinogenic, antiatherosclerotic, antithrombotic, antimicrobial, antiinflammatory and antioxidant effects [5,6,7,8,9]. Recently the scientific community has taken it seriously and started to conduct proper investigation and clinical trials to confirm these activities. Indeed for its antioxidant properties the role of garlic in preventing age-related diseases and cardiovascular disease has been investigated extensively over the past 10–15 years. The more important clinical application is its role as an antihypertensive and protector of the cardiovascular system. Epidemiological studies showed an inverse correlation between garlic consumption and the progression of cardiovascular disease. Cardiovascular disease is associated with multiple factors (raised serum total cholesterol, raised LDL and an increase in LDL oxidation, increased platelet aggregation and hypertension) and numerous *in vitro* studies have confirmed the ability of garlic to reduce these parameters [10,11]. However the numerous experimental findings are frequently conflicting. The negative results obtained in some clinical trials may also have originated from the use of different garlic preparations, unknown active components and their bioavailability, inadequate randomization, selection of inappropriate subjects and the short duration of trials [12]. Nevertheless, garlic appears effective in reducing parameters associated with cardiovascular disease, even if more in-depth and appropriate studies are required. The beneficial effects of garlic supplementation in reducing blood pressure and offering cardioprotection seems to be due to its ability to counteract oxidative stress [13]. The antioxidant activity of *Allium* spp. has been attributed mainly to a variety of sulphur-containing compounds and their precursors [14,15,16]. Scientific evidence shows that allicin, diallyl disulphide and diallyl trisulphide appeared to be the main antioxidative compounds [17,18]. In addition, the antioxidant activity is also related to other bioactive compounds: dietary fibers, microelements (especially Se) and polyphenols [19,20]. The antioxidant activity of some wild *Allium* species has been analysed in the Republic of Serbia, in Russia, in Japan and in other countries [14,21,22] but no information is available on the *in vitro* antioxidant activities of Italian *Allium* species growing wild. The aim of this review is to understand the mechanism of action and therapeutic efficacy of *Allium*.

## 2. Mechanism of Antioxidant Action of *Allium*

Nencini *et al.* [23] investigated the *in vitro* antioxidant activity of aged (up to 20 months) 15% hydroethanolic extracts of different parts (bulbs, bulblets, flower bulblets, flowers, and leaves) of three *Allium* spontaneous species which are endemic to the Italian flora: *Allium neapolitanum* Cyr., *Allium subhirsutum* L., *Allium roseum* L. and compared it with the *in vitro* antioxidant activity of aged 15% hydroethanolic extracts of bulbs and leaves of garlic. The aged extracts obtained from the leaves showed the best antioxidant activity, followed by flowers and then by bulbs in both used tests, while flower bulblets and bulblets exhibited lower results or no activity. The polyphenol content was generally directly correlated with antioxidant/antiradical activity. This study confirms the data obtained in previous researches, the wild-type species of *Allium* and in particular organs other than bulbs are more active and efficacious than garlic bulb. In conclusion, although Nencini *et al*. [23] did not detect any organosulphur compound in the hydroethanolic extract, this study increases the knowledge on possible uses of garlic and of Italian *Allium* wild species, today almost neglected as food or as source of potential medicinal agents [23].

Nencini *et al.* [24] also investigated the protective effect on liver injury induced by ethanol in rats of *Allium neapolitanum* Cyr., a spontaneous Italian flora species, compared with garlic (*Allium sativum* L.). Male albino Wistar rats were orally treated with fresh *Allium* homogenates (leaves or bulbs, 250 mg/kg) daily for 5 days, whereas controls received vehicle only. At the end of the 5-day experimental period, the animals received an acute ethanol dose (6 mL/kg, i.p.) 2 h before the last *Allium* administration and were sacrificed 6 h after ethanol administration. The activities of catalase (CAT), superoxide dismutase (SOD), and glutathione reductase (GR) and the levels of malondialdehyde (MDA), ascorbic acid (AA), and reduced (GSH) and oxidized glutathione in liver tissue were determined. Administration of both *Allium* species for 5 days (leaves or bulbs) led to no statistical variation of nonenzymatic parameters *versus* the control group; otherwise *Allium* treatment caused an increase of GSH and AA levels compared with the ethanol group and a diminution of MDA levels, showing in addition that *A. neapolitanum* bulb had the best protective effect. Regarding the enzymatic parameters, GR and CAT activities were enhanced significantly compared with the ethanol group, whereas SOD activity showed a trend different from other parameters estimated. However, the treatment with both *Allium* species followed by acute ethanol administration reestablished the nonenzymatic parameters similar to control values and enhanced the activities of the enzymes measured. These results suggest that fresh *Allium* homogenates (leaves or bulbs) possess antioxidant properties and provide protection against ethanol-induced liver injury [24].

Another study performed by Park *et al.* [25] describes the antioxidant activities and antigenotoxic effects of garlic extracts prepared by different processing methods. Aged-garlic extract (AGE) showed a significantly higher total phenolic content (562.6 ± 1.92 mg/100 g garlic acid equivalents) than those of raw garlic extract (RGE) or heated garlic extract (HGE). The SC_50_ for DPPH RSA in HGE was significantly the highest at 2.1 mg/mL. The SC_50_ for SOD-like activity in garlic extracts was, in decreasing order, RGE (7.3 mg/mL) > AGE (8.5 mg/mL) > HGE (9.2 mg /mL). The ED_50_ of AGE was the highest (19.3 μg/mL) regarding H_2_O_2_ induced DNA damage and its inhibition rate was 70.8%. The ED_50_ of RGE for 4-hydroxynonenal (a lipid peroxidation product)-induced DNA damage was 38.6 μg/mL, followed by AGE > HGE. Although the heat treatment of garlic tended to decrease the TPC and SOD-like activity and increased DPPH RSA, garlic, in general, has significant antioxidant activity and protective effects against oxidative DNA damage regardless of processing method.

In conclusion, heating after grinding produces a reduction in the amount of total phenolics in the garlic, probably owing to its biodegradation at high temperature. Raw and aged garlic contains high comparable quantities of total phenolic and possess high antigenotoxic effect. It is suggested that a proper heat treatment and aged-processing could be used to enhance the amount of bioactive compounds and antioxidant capacity of garlic. Garlic extracts exhibit significant protective effects against DNA damage induced by H_2_O_2_ and HNE, which might be related to antioxidant activity [25].

In another study Lawal *et al.* [26] evaluated the antioxidant efficacy of heated garlic juice (HGJ) in liver with that of ascorbic acid (AA) in rats exposed to acute dose of cadmium (4 mg·kg^−1^ bd. wt). The rats were either given HGJ (100 mg·kg^−1^ bd. wt) orally, daily for 4 weeks or AA (100 mg·kg^−1^ bd. wt) orally, daily for 4 weeks or both or cadmium (4 mg·kg^−1^ bd. wt) intraperitoneally for 3 days. Another group of rats was given cadmium (4 mg·kg^−1^ bd. wt) intraperitoneally for 3 days after pretreatment with either HGJ (100 mg·kg^−1^ bd. wt) or ascorbic acid (100 mg·kg^−1^ bd. wt) for 4 weeks and the liver excised. The results obtained show that AA and HGJ significantly reduced the level of liver malondialdehyde (MDA) induced by cadmium compared to control (*p* < 0.05) but AA tends to be more potent when compared with HGJ. The presence of either HGJ or AA also significantly reduced the levels of ROS in the presence of cadmium (Cd). The presence of either AA or HGJ pre-treatment produced significant increase in liver SOD and catalase activities when compared with rats treated with Cd alone. There was no significant reduction in the activities of these enzymes in the presence of cadmium compared to control. Western blots showed that the expressions of Nrf2 and NQO1 in the liver were significantly increased by 3 and 1.7-fold, respectively, in the AA pretreated mice when compared with Cd. However no significant changes were seen in HGJ pretreated rats. The expression of HO-1 was not significantly increase in the AA pretreated rats. The results show that though both ascorbic acid and HGJ are efficient in preventing Cd-induced damage in the rat liver, ascorbic acid appeared to be a more powerful antioxidant than heated garlic juice in preventing cadmium-induced oxidative damage in liver and its action may be mediated in parts via Nrf2-keap1 pathway [26].

Cytoprotective effects of chemopreventive agents may be attributed to the induction of antioxidant enzymes. Among these, the induction of glutamate-cysteine ligase (GCL) protects cells from oxidative injury by increasing glutathione (GSH) content. Nuclear factor erythroid-2-related factor 2 (Nrf2) transcriptionally regulates the expression of genes encoding for GCL and other cysteine-metabolizing enzymes. Despite extensive studies on the components in garlic, little information is available on organosulfur by-products made from garlic. In this respect, Kay *et al.* [27] investigated whether ajoene, a chemically stable garlic by-product, has the ability to activate Nrf2 and induce GCL, and, if so, what is the role of activating Nrf2 in cytoprotection against oxidative stress. Immunoblotting and reporter gene assays were performed in HepG2 cells. Ajoene treatment activated Nrf2, as indicated by increased phosphorylation and nuclear accumulation of Nrf2, decreased interaction with Kelch-like ECH-associated protein-1, and decreased Nrf2 ubiquitination. Consistently, treatment of ajoene increased antioxidant response element reporter gene activity and the mRNA and protein levels of GCL subunits. Ajoene activated protein kinase C-delta (PKCdelta). Inhibition of PKCdelta activation by rottlerin abrogated its ability to activate Nrf2 and induce GCL, suggesting that ajoene promotes the Nrf2-dependent antioxidant defense system via PKCdelta activation. Consequently, ajoene prevented cell death, GSH depletion, and hydrogen peroxide production elicited by *tert*-butyl hydroperoxide. The important role of Nrf2 in cytoprotection was verified by the reversal of ajoene’s ability to protect hepatocytes in Nrf2-knockout mice. These results demonstrate that ajoene increases PKCdelta-dependent Nrf2 activation, GCL induction, and the cellular GSH concentration, which may contribute to protecting cells from oxidative stress [27].

Identification of agents that are nontoxic but can delay onset and/or progression of breast cancer, which is the main leading cause of cancer-related deaths among women, is highly desirable. Garlic-derived organosulfur compounds (OSCs) have highly effective antitumor effects, but the mechanism has yet to be investigated. Das *et al.* [28] examined the effect of diallyl trisulfide (DATS), a promising cancer chemopreventive constituent of garlic, on growth of two cell lines respectively, MCF-7 human breast cancer cells and nontumorigenic MCF-12a mammary epithelial cells. The effects of DATS were examined by MTT assay, clonogenic survival assay, ELISA based apoptotic assay, TUNEL assay, immunofluoresence staining, flow cytometry, RT-PCR and western blot analysis. Garlic constituent diallyl trisulfide (DATS) suppresses viability of cultured MCF-7 and MCF-12a cells, respectively, by decreasing the percent of cells in G(2)/M phase and inducing apoptotic cell death. DATS-induced apoptosis was markedly elevated in MCF-7 cells compared with MCF-12a cells and this was correlated with elevated levels of cyclin B1. The results from semi-quantitative and real-time RT-PCR indicated that DATS-enhanced the expression levels of FAS and cyclin D1, but in contrast, downregulated the expression levels of Akt and Bcl-2. Furthermore, the DATS-induced apoptosis was correlated with induction of pro-apoptotic Bax protein and p53 protein expression was upregulated and translocation to nucleus in MCF-7 cells. Together, the results of the present study show, for the first time, that DATS administration might offer a novel strategy for the treatment of human breast cancer [28].

**Figure 2:**
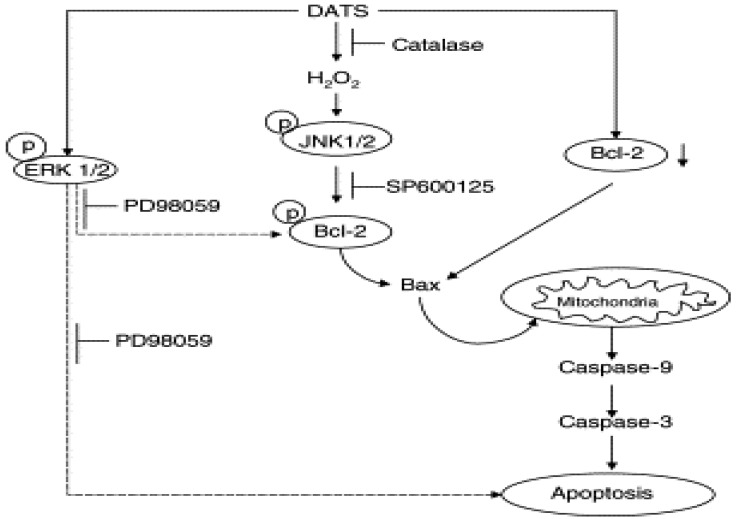
Proposed mechanism by diallyl trisulfide against carcinogenesis.

Garlic-derived organosulfur compounds such as diallyl sulfide (DAS), diallyl disulfide (DADS), and diallyl trisulfide (DATS) provide significant protection against carcinogenesis (Figure 2). Treatment of glioblastoma cells with garlic compounds triggered production of ROS that induced apoptosis with the phosphorylation of p38 MAPK and activation of the redox-sensitive JNK1 pathway. Pretreatment of cells with ascorbic acid attenuated ROS production, p38 MAPK phosphorylation, and JNK1 activation [29]. Pretreatment with JNK1 inhibitor I also significantly reduced cell death. Increases in intracellular free [Ca(2+)], expression of calreticulin, and activation of caspase-4 indicated involvement of endoplasmic reticulum (ER) stress in apoptosis. Other events in apoptosis included overexpression of Bax, down-regulation of Bcl-2 and some BIRC proteins, mitochondrial release of cytochrome c and Smac into the cytosol, and activation of calpain, caspase-9, and caspase-3. Garlic compounds induced apoptosis in glioblastoma cells due toproduction of ROS, increase in ER stress, decrease in Delta psi(m), and activation of stress kinases and cysteine proteases [29].

Allicin, a major ingredient of fresh garlic extract that is produced during the crushing of garlic cloves, exerts various beneficial biological effects, including a broad spectrum of antimicrobial activity, antihyperlipidaemic and antihypertensive effects. However, how allicin affects the immune system is less well known, and its effect on human T cells has never been studied. Sela *et al.* [30] examined the *in vitro* effects of allicin on the functioning of T cells related to their entry to inflamed extravascular sites. They found that allicin (20–100 microm) inhibits the SDF-1alpha (CXCL12)-induced T cell migration through fibronectin (FN), and that this inhibition is mediated by the down-regulation of (i) the reorganization of cortical actin and the subsequent T cell polarization, and (ii) T cell adhesion to FN. Moreover, allicin also inhibited T cell adhesion to endothelial cells and transendothelial migration [30]. The mechanisms underlying these inhibitory effects of allicin are associated with its ability to down-regulate the phosphorylation of Pyk2, an intracellular member of the focal adhesion kinases, and to reduce the expression of the VCAM-1- and FN-specific alpha4beta1-integrin (VLA-4). The ability of allicin to down-regulate these chemokine-induced and VLA-4-mediated T cell functions explains its beneficial biological effects in processes where T cells play an important role and suggests that allicin may be used therapeutically with chronic inflammatory diseases [30].

## 3. Therapeutic Efficacy of *Allium*

People have more and more concerned about allitridum as studies have shown that taking more raw garlic associated with a lower risk for cancers of the alimentary system. Li *et al.* [31] tried to examine whether a large dose of allitridum and a microdose of selenium prevent gastric cancer. A double-blind intervention study was performed on the participants aged (35–74) years, who had matched at least one of the following criteria: (1) a medical history of stomach disorder, (2) a family history of tumours, or (3) smoking and/or alcohol consumption [31]. A total of 2,526 and 2,507 persons were randomly enrolled into the intervention group and control group, respectively, from 288 natural villages of seven communities in Qixia County, Shandong Province, China. Each person of the intervention group orally took 200 mg synthetic allitridum every day and 100 μg selenium every other day for one month of each year during November 1989 to December 1991. At the same time, people in control group were given two placebo capsules containing corn oil with the identical appearance to that in the intervention group [31]. For all subjects the large dose of allitridum was accepted and no harmful side effects were found during the study. In the first follow-up five years (1992–1997) after stopping the intervention, the morbidity rates of malignant tumours in the intervention group declined by 22%, in contrast to the control group, declined by 47.3% [31]. After adjusting for age, gender, and other potential confounders, relative risks (RRs) for all tumours and gastric cancer of the whole population were 0.67 (95% CL: 0.43–1.03) and 0.48 (95% CL: 0.21–1.06), respectively, and for male group they were 0.51 (95% CL: 0.30–0.85) and 0.36 (95% CL: 0.14–0.92), respectively. No significant protective effect was found for the female subgroup. The present study proves that large doses of allitridum and microdorse of selenium may effectively prevent gastric cancer, especially in men [31].

Another randomized trial has yielded mixed results on the effects of treatment for *Helicobacter pylori* and little information on the effects of vitamins or garlic supplements on precancerous gastric lesions [32]. You *et al.* [32] conducted a randomized trial to test the effects of one-time *H. pylori* treatment and long-term vitamin or garlic supplements in reducing the prevalence of advanced precancerous gastric lesions. Most of the adults aged 35–64 years in 13 randomly selected villages in Linqu County, Shandong Province, China, were identified and given baseline endoscopies in 1994. In 1995, 3,365 eligible subjects were randomly assigned in a factorial design to three interventions or placebos: amoxicillin and omeprazole for 2 weeks in 1995 (*H. pylori* treatment); vitamin C, vitamin E, and selenium for 7.3 years (vitamin supplement); and aged garlic extract and steam-distilled garlic oil for 7.3 years (garlic supplement) [32]. Subjects underwent endoscopies with biopsies in 1999 and 2003, and the prevalence of precancerous gastric lesions was determined by histopathologic examination of seven standard biopsy sites [32]. The 3,365 eligible randomized subjects represented 93.5% of those with baseline endoscopy and included all baseline histologic categories except gastric cancer [32]. Only 0.18% had normal gastric mucosa. Logistic regression was used to estimate the intervention effects on the odds of advanced precancerous gastric lesions, and *t*-tests were used to assess effects on histologic severity. All statistical tests were two-sided. *H. pylori* treatment resulted in statistically significant decreases in the combined prevalence of severe chronic atrophic gastritis, intestinal metaplasia, dysplasia, or gastric cancer in 1999 (odds ratio [OR] = 0.77; 95% confidence interval [CI] = 0.62 to 0.95) and in 2003 (OR = 0.60; 95% CI = 0.47 to 0.75), and had favorable effects on the average histopathologic severity and on progression and regression of precancerous gastric lesions in 2003. *H. pylori* treatment did not reduce the combined prevalence of dysplasia or gastric cancer. However, fewer subjects receiving *H. pylori* treatment (19/1130; 1.7%) than receiving placebo (27/1128; 2.4%) developed gastric cancer (adjusted *p* = 0.14). No statistically significant favorable effects were seen for garlic or vitamin supplements. *H. pylori* treatment reduces the prevalence of precancerous gastric lesions and may reduce gastric cancer incidence, but further data are needed to prove the latter point. Long-term vitamin or garlic supplementation had no beneficial effects on the prevalence of precancerous gastric lesions or on gastric cancer incidence [32].

Epidemiological and animal studies suggest AGE and its organosulfur constituents, such as *S*-allylcysteine and *S*-allylmercaptocysteine have anticarcinogenic effects. To confirm these effects in humans, a preliminary double-blind, randomized clinical trial using high-dose AGE (AGE 2.4 mL/day) as an active treatment and low-dose AGE (AGE 0.16 mL/day) as a control was performed on patients with colorectal adenomas-precancerous lesions of the large bowel [33]. The study enrolled 51 patients who were diagnosed as carrying colorectal adenomas. The patients were randomly assigned to the two groups after adenomas larger than 5 mm in diameter were removed by polypectomy. The number and size of adenomas right before intake (0 month) and at 6 and 12 month after intake were measured using colonoscopy [33]. Thirty-seven patients (19 in the active group, 18 in the control group) completed the study and were evaluated for the efficacy of AGE. The number of adenomas increased linearly in the control group from the beginning (the baseline), but AGE significantly suppressed both the size and number of colon adenomas in patients after 12 month of high-dose treatment (*p* = 0.04). The results suggest AGE suppresses progression of colorectal adenomas in humans. It appears that AGE has multiple pathways to reduce cancer incidence and suppress its growth and proliferation [33].

Although the therapeutic role of ajoene, an organosulfur compound of garlic, in cardiovascular diseases and mycology has been established, its usefulness in cancer treatment has only recently been suggested. Tilli *et al.* [34] applied ajoene topically to the tumors of 21 patients with either nodular or superficial basal cell carcinoma (BCC). A reduction in tumor size was seen in 17 patients. Immunohistochemical assays for Bcl-2 expression in a selection of these tumors before and after treatment showed a significant decrease in this apoptosis-suppressing protein [34]. On average, the percentage of tumor cells expressing the proliferation marker Ki-67 was not decreased, which suggests that the action of ajoene is not explained by a cytostatic effect. To obtain further insight into the mode of action of ajoene, the BCC cell line TE354T and a short-term primary culture of BCC were analyzed for apoptosis induction after treatment with the drug [34]. Apoptosis was detected by morphology of the cells and by flow cytometry. Ajoene induced apoptosis in a dose- and time-dependent manner in these cultures. Taking together the results of the *in vivo* and *in vitro* studies, Tilli *et al.* [34] concluded that ajoene can reduce BCC tumor size, mainly by inducing the mitochondria-dependent route of apoptosis [34].

## 4. Conclusions

In conclusion, garlic has been found to contain a large number of potent bioactive compounds with anticancer properties, largely allylsulfide derivatives. Garlic derivatives have been found to influence an increasing number of molecular mechanisms in carcinogenesis, including DNA adduct formation, scavenging of free radicals, mutagenesis, cell proliferation and differentiation, and angiogenesis. The growth rate of cancer cells is reduced by garlic, with cell cycle blockade that occurs particularly in the G2/M phase. Apoptosis is stimulated by garlic [35].

Stimulation of apoptosis has been related to inactivation of the Akt signaling axis. Rodent recipients of either androgen-sensitive or androgen-insensitive human prostate cancer cell lines show reduction in the serum concentration of prostate specific antigen [35].

In addition to inhibiting primary cancer, allium derivatives from garlic may further inhibit metastatic processes. In an androgen-independent prostate cancer mouse model, the water-soluble allium derivative, *S*-allyllmercaptocysteine, inhibited metastases to the lung and adrenal gland by 90%.

Furthermore, garlic is a seleniferous plant, accumulating selenium from the soil against a concentration gradient. Selenium has many anticancer actions, particularly in control of genes involved in carcinogenesis [35].
